# Psychological interventions for people with psychotic experiences: protocol for a systematic review and meta-analysis

**DOI:** 10.1186/s13643-019-1041-5

**Published:** 2019-05-23

**Authors:** Emma Soneson, Debra Russo, Clare Knight, Louise Lafortune, Margaret Heslin, Jan Stochl, Alex Georgiadis, Julieta Galante, Robbie Duschinsky, Nick Grey, Leticia Gonzalez-Blanco, Juliet Couche, Michelle Griffiths, Hannah Murray, Nesta Reeve, Joanne Hodgekins, Paul French, David Fowler, Sarah Byford, Mary Dixon-Woods, Peter B. Jones, Jesus Perez

**Affiliations:** 1 Department of Psychiatry, University of Cambridge, Cambridge, CB2 0SZ UK; 2 Department of Public Health and Primary Care, Cambridge Institute of Public Health, University of Cambridge, Cambridge, CB2 0SR UK; 3 King’s College London, Institute of Psychiatry, Psychology & Neuroscience, De Crespigny Park, London, SE5 8AF UK; 4 THIS Institute (The Healthcare Improvement Studies Institute), University of Cambridge, Cambridge, CB2 0AH UK; 5 The Primary Care Unit, Cambridge Institute of Public Health, University of Cambridge School of Clinical Medicine, Box 113, Cambridge Biomedical Campus, Cambridge, CB2 0SR UK; 6 Hove, UK; 7 Department of Psychiatry, University of Oviedo - CIBERSAM - Servicio de Salud del Principado de Asturias, Oviedo, Spain; 8 Health in Mind, Sussex Partnership NHS Foundation Trust, Woodside, The Drive, Hellingly, East Sussex, BN27 4ER UK; 9 Elizabeth House, Fulbourn Hospital, Cambridge, CB21 5EF UK; 10 Oxford Centre for Anxiety Disorders and Trauma, Paradise Square, Oxford, OX1 1TW UK; 11 Norfolk and Suffolk NHS Foundation Trust, Hellesdon Hospital, Drayton High Road, Norwich, NR6 5BE UK; 12 Department of Clinical Psychology, Elizabeth Fry Building, Norwich Medical School, University of East Anglia, Norwich, NR4 7TJ UK; 13 Psychosis Research Unit, Greater Manchester Mental Health NHS Foundation Trust, Manchester, M25 3BL UK; 14 School of Psychology, Pevensey Building, University of Sussex, Brighton, BN1 9QH UK

**Keywords:** Psychosis, Ultra-high risk, At-risk mental state, Psychotic experiences, Psychological intervention, Therapy, Cognitive behavioural therapy

## Abstract

**Background:**

Many people who have common mental disorders, such as depression and anxiety, also have some psychotic experiences. These experiences are associated with higher clinical complexity, poor treatment response, and negative clinical outcomes. Psychological interventions have the potential to improve outcomes for people with psychotic experiences. The aims of this systematic review are to (1) synthesise the evidence on the effectiveness and cost-effectiveness of psychological interventions to reduce psychotic experiences and their associated distress and (2) identify key components of effective interventions.

**Methods:**

Our search strategy will combine terms for (1) psychological interventions, (2) psychotic experiences, and (3) symptoms associated with psychotic experiences. We will search the following online databases: MEDLINE, Embase, PsycINFO, all Cochrane databases, British Nursing Index (BNI), Cumulative Index to Nursing and Allied Health Literature (CINAHL), Health Management Information Consortium (HMIC), Education Resources Information Center (ERIC), and EconLit. Our primary outcome is the proportion of people who recovered or remitted from psychotic experiences after the intervention. Our secondary outcomes are changes in positive psychotic symptoms, negative psychotic symptoms, depression, anxiety, functioning (including social, occupational, and academic), quality of life, and cost-effectiveness. Two independent reviewers will judge each study against pre-specified inclusion and exclusion criteria and will extract study characteristics, outcome data, and intervention components. Risk of bias and methodological quality will be assessed using the Effective Public Health Practice Project Quality Assessment Tool for Quantitative Studies and the Drummond Checklist. Results will be synthesised using random-effects meta-analysis and narrative synthesis.

**Discussion:**

The identification of effective psychological interventions and of specific components associated with intervention effectiveness will augment existing evidence that can inform the development of a new, tailored intervention to improve outcomes related to psychotic symptoms, anxiety and depression, distress, functioning, and quality of life.

**Systematic review registration:**

PROSPERO CRD42016033869

## Background

Many people who have common mental disorders (i.e. depressive and anxiety disorders) also have some psychotic experiences such as attenuated paranoia or voice hallucinations. Findings from large population studies have shown that these psychotic phenomena co-occur with depression and anxiety [1]. In fact, psychosis, depression, and anxiety share mechanisms [2–7] and causes, including childhood and later trauma [8–10]. Longitudinal clinical data indicate that psychotic experiences during adolescence or young adulthood may be associated with a range of non-psychotic mental disorders later in life [11]. More generally, psychotic experiences are associated with higher clinical complexity, poor response to treatment [12–14], bad clinical and functional outcomes, and increased risk of self-harm [15–19].

This evidence suggests that psychotic experiences may be useful markers of severity in common mental disorders [4] in addition to serving as indicators of possible transition to psychotic illness. Recent research has found that most people with common mental disorder with psychotic experiences, regardless of whether they receive specialised psychological treatment, rarely develop frank psychotic disorders [15, 20]. This suggests that common mental disorder with psychotic experiences may represent a distinct form of illness that might benefit from targeted therapeutic approaches. A reasonable hypothesis is that recovery rates in this population might be improved by focusing on psychotic experiences and their associated distress and not exclusively on reducing transitions.

The first challenge is the accurate identification and treatment of common mental disorder with psychotic experiences. Current psychiatric diagnostic classifications [21, 22] do not acknowledge the presence of psychotic experiences in more common depressive or anxiety disorders. Indeed, very few clinical measurement scales for common psychiatric morbidity include psychotic experiences [1]. These experiences do not necessarily reach the threshold for treatment in specialised secondary care services in the first instance and often remain undetected in more general health care settings. For example, people with common mental disorders in England are usually treated in primary care; most are referred to Improving Access to Psychological Therapies (IAPT) services, also called Psychological Wellbeing Services, which were developed to enable more people with common mental disorders to access talking therapies. These services treat more than 1,000,000 people a year, providing steps 2 and 3 in the four-step model of care following the National Institute for Health and Care Excellence (NICE) guidelines for depression [23] and anxiety and related disorders [24]. They mostly offer standard cognitive behavioural therapy (CBT) for people aged 16 to 65. As in other primary care settings, psychotic experiences are not routinely measured in the IAPT population nor taken into account in therapy [25]. Yet, within IAPT, psychotic experiences are relatively common; results from a recent study showed that approximately 30% of individuals in step 3 reported having these experiences [25]. Also, psychotic experiences were associated with higher depression and anxiety scores before and after the initial period of therapy, indicating poor recovery [25]. This suggests strongly that people with psychotic experiences may fall into a service gap because their symptoms neither reach the increasingly high threshold for secondary care, nor are they explicitly and adequately addressed by standard psychological therapies.

To date, the psychological intervention for addressing psychotic experiences that has been most investigated is CBT [26]. Other types of psychological therapies and interventions (e.g. mindfulness interventions, behaviour therapy) for psychotic experiences are less frequently studied [27]. Additionally, while psychological therapy has been shown to reduce psychotic experiences [28–30], less is known about its effects on other symptoms such as distress, sleep, social interaction, or functioning. These factors are important to the individual and can act as personal indicators that personal, social, and clinical recovery have been achieved [31–33]. Further, a detailed account of the components included in successful interventions could be useful for tailoring a practical therapeutic toolkit for people with common mental disorder with psychotic experiences. However, the contribution of specific components to the effectiveness of psychological interventions is under-studied. A comprehensive review of the literature is required.

## Methods

### Aim

The aim of this systematic review is to provide an evidence synthesis of the effectiveness and cost-effectiveness of all psychological interventions to reduce psychotic experiences and the associated distress through meta-analysis, supported by narrative synthesis where appropriate. To this end, the proposed systematic review will answer the following questions:

1. Which psychological interventions have been effective for the treatment of psychotic experiences and have improved outcomes related to psychotic symptoms, symptoms of depression and anxiety, distress, functioning (including social, occupational, and academic), and quality of life?
2. What are the common components of psychological interventions for psychotic experiences that improve outcomes related to psychotic symptoms, symptoms of depression and anxiety, distress, functioning (including social, occupational, and academic), and quality of life?
3. Which psychological interventions used for the treatment of psychotic experiences are cost-effective?

In fulfilling these aims, this review will provide an overview of psychological interventions for psychotic experiences and give recommendations for the treatment of these experiences and their associated distress. Additionally, it will contribute to the development of a tailored intervention for use within IAPT services as part of the NIHR Programme Grant for Applied Research (RP-PG-0616-20003): Tailoring evidence-based psychological therapY for People with common mental disorder including Psychotic EXperiences (TYPPEX).

The review will follow the PRISMA guidelines [34], and this protocol will adhere to the PRISMA-P protocol guidance [35]. The review has been registered in the PROSPERO International Prospective Register of Systematic Reviews (registration number: CRD42016033869; 22 May 2018 version).

### Population

#### Participant characteristics

We will include studies of adolescents and adults aged 16 years and older in order to reflect the ages when most of these clinical experiences tend to appear and become more clinically distinctive [36] and to focus on interventions that may be applied in adult mental health settings, such as IAPT services, with no restrictions on sex, gender, or ethnicity. If studies include participants younger than 16 and do not separate outcome data by age, we will only include those studies where the mean age of the participants is ≥ 16.

#### Diagnosis

Our condition of interest is psychotic experiences as measured by a valid and reliable measure (clinician rated *or* self-report). As different authors use diverse terms to describe these experiences, we will also include studies whose interventions focused on the following diagnoses: at-risk mental state, attenuated psychosis, psychosis-like experiences, unusual experiences, sub-threshold psychosis, prodromal psychosis, schizotypal disorders, psychotic depression, and psychotic anxiety. We will further include interventions for people at ultra-high risk (UHR) and clinical high risk (CHR) for psychosis. We will not include studies focused on populations with diagnoses of psychosis, first episode psychosis (FEP), schizophrenia, or bipolar disorder.

We will further include studies where patients have depression, anxiety, and/or substance use in addition to their psychotic experiences, due to their high prevalence in this population. We will exclude studies focused on participants with psychotic experiences and any of the following: intellectual disability, epilepsy, dementia, Alzheimer’s disease, Parkinson’s disease, and Huntington’s disease. The purpose of this exclusion criterion is to control for confounding in the assessment of intervention effect.

#### Intervention

We will include studies that focus on psychological and psychosocial therapies or interventions. Using Cox and colleagues’ 2014 review as guidance [37], we have included all psychological interventions as divided into cognitive behavioural therapy, integrative therapy, humanistic therapy, and psychodynamic therapy. Common examples of psychological therapies are presented below, with their main elements listed after:

- Cognitive behavioural therapy (CBT)—cognitive restructuring and behavioural change
- Behavioural therapy (BT)—focuses on learned behaviours and how the context or environment influences those behaviours
- Cognitive therapy (CT)—cognitive restructuring
- Mindfulness therapy—focusing on and attending to present experiences
- Interpersonal therapy (IPT)—improving social relationships and social skills
- Problem-solving therapy (PST)—identifying and creating solutions to current problems
- Play therapy (PT)—increasing participation in activities
- Humanistic therapy (HT)—supportive, empathetic therapy without judgement or advice
- Psychodynamic therapy (PDT)—interpretation and transference to resolve unconscious conflict

We will include studies whose interventions provide other relevant psychological interventions not listed above (please see search strategy item S4 for more detail on psychological interventions included in the search.)

We will exclude studies in which psychiatric medication was provided *as part of the intervention protocol*. We have not placed a restriction on the proportion of participants taking medication for depressive or anxiety disorders, but will exclude studies in which greater than 25% of participants received antipsychotics due to clinical need. This exclusion criterion aims to limit confounding due to the use of antipsychotic medication.

We have placed no restriction on intervention providers (e.g. clinical psychologist vs. psychiatrists), duration of intervention, or mode of delivery (e.g. online vs. face-to-face).

#### Comparators

For inclusion in the meta-analysis, we will require studies to have a comparator, which may be another type of psychological intervention or treatment as usual, but which must not be psychopharmacological/dietary in nature. We will also include studies without a comparator (e.g. pre-post studies, cohort studies) within our narrative synthesis. The field of psychological therapy for psychotic experiences is still relatively new, and although some therapy frameworks may currently be at preliminary stages of investigation (i.e. not ready for evaluation in controlled trials), they are still of interest to our review. We have not placed any restrictions on comparators in relation to cost-effectiveness.

#### Outcomes

Our primary outcome of interest is the proportion of participants achieving symptomatic recovery or remission of psychotic experiences, as determined by validated clinician-rated or self-report measures (e.g. the Comprehensive Assessment of At-Risk Mental States (CAARMS) [38]). Secondary outcomes of interest include changes in psychotic symptoms, distress, anxiety and depressive symptoms, functioning (including social, occupational, and academic functioning), and quality of life. These outcome measures were chosen because of their potential to inform psychological interventions to improve health outcomes for people with psychotic experiences. We will further include studies that report economic outcomes (broadly defined), including costs, resource use, employment impacts, lost productivity, quality-adjusted life years (QALYs), or disability-adjusted life years (DALYs). Economic data will be included to determine whether these interventions are economically viable options for implementation in current services.

We will exclude studies that solely report on the binary outcome of conversion or non-conversion to psychotic disorders. As stated above, the rationale behind this exclusion lies in the low rates of transition from having psychotic experiences to frank psychotic disorder (estimated at approximately 30% over a 2-year follow-up period) [39]. Furthermore, the therapy that will be designed for the TYPPEX programme does not seek to prevent transition, but rather to provide symptomatic recovery, and measures of transition do not explicitly report on symptomatic recovery or improvement.

#### Setting

We have not placed any restriction on setting.

#### Study design

We will include all experimental study designs that provide data on our primary and/or secondary outcomes, as measured by a validated and reliable tool, including but not limited to randomised controlled trials (RCTs), controlled clinical trials (CCTs), and pre-post designs.

We will not include qualitative studies because they cannot adequately address our aims. We will further exclude the following publication types: books, letters, trade articles, magazines, opinions/editorials, replies/commentaries, posters, conference abstracts (in absence of full texts), reviews, protocols, guidelines, service evaluations, and case studies.

#### Miscellaneous exclusion criteria

We will exclude papers from before the year 2000, which was when the at-risk mental state [38] became widely adopted. We have no exclusion criteria for publication language, as long as an English abstract is provided.

### Information sources

We will search the following online databases, with an end date of 15 December 2018: MEDLINE, Excerpta Medica DataBase (Embase), PsycINFO, all Cochrane databases, British Nursing Index (BNI), Cumulative Index to Nursing and Allied Health Literature (CINAHL), Health Management Information Consortium (HMIC), Education Resources Information Center (ERIC), and EconLit. We will further hand search reference lists of relevant reviews and journals for additional citations. We will also search the World Health Organization International Clinical Trials Registry Platform (WHO ICTRP) for relevant trials. For trials that have not published results 30 months after their anticipated completion date, we will contact authors to provide data. We will further search the following grey literature databases for dissertations and conference papers: PsycINFO, CINAHL, Google Scholar, EThOS, and Open Grey.

### Search strategy

The search strategies for each of the databases were developed in partnership with experts at the University of Cambridge Medical Library. Please see the Appendix for the search strategy that will be entered in PsycINFO, CINAHL, and ERIC (all accessed via EBSCO). We did not use any peer review service for our search strategy (e.g. Peer Review of Electronic Search Strategies, PRESS).

### Study records

#### Data management

EndNote X8 will be used to manage records throughout the review process to identify and remove duplicates, obtain full texts, and categorise papers according to inclusion or exclusion.

#### Study selection

After removal of duplicate citations, studies will be screened in two stages: title/abstract and full text. DR and ES will complete title and abstract screening and full text screening independently using the inclusion and exclusion criteria described above. DR and ES will discuss all disputed papers, and a senior team member (JP) will resolve any discrepancy. Where there is not sufficient data to determine inclusion or exclusion, protocols for that study will be consulted if available, and study authors will be contacted to request any data missing from methods or results. For each stage of screening, we will calculate Cohen’s kappa to measure inter-rater agreement. We have defined kappa quality as follows: values ≤ 0 as no agreement, 0.01–0.20 as no to slight agreement, 0.21–0.40 as fair agreement, 0.41–0.60 as moderate agreement, 0.61–0.80 as substantial agreement, and 0.81–1.00 as nearly perfect agreement [40]. In the event of a poor Cohen’s kappa, we will have a third reviewer review all papers selected for full-text review and will triangulate using this third opinion.

#### Data extraction

Two trained research assistants (DR and ES) will extract data from the included studies. All extraction and components tables will be piloted with a small sample of papers (*N* = 5) before they are used for the review and will be amended as necessary after piloting. The reviewers will independently extract study characteristics, outcome data, and intervention components, and JP will resolve any discrepancies not solved through discussion between DR and ES. Again, where variables of interest are not clearly presented in the studies, intervention protocols will be referenced and/or study authors contacted.

#### Study and sample characteristics

In terms of general study characteristics, we will extract the following information:

- Author
- Location
- Study design
- Sample size
- Sample characteristics (age, sex, ethnicity, etc.)
- Participation and attrition rates

#### Outcome data

To measure the effectiveness of the intervention in terms of our primary and secondary outcomes, we will extract the following:

- Which outcomes were used (primary and secondary)
- Primary criterion used to determine at-risk mental state
- Tools used to measure outcomes
- Terminology used to represent psychotic experiences (i.e. diagnosis)
- Duration of follow-up measurement
- Baseline symptom severity for all outcomes
- Findings/outcomes (only those of interest in our systematic review will be extracted) as measured by authors

#### Intervention components

We will also extract all available intervention components from each of the studies, regardless of intervention outcome. We will extract information both inherent to the intervention (e.g. characteristics of the intervention or therapy itself) as well as information about its implementation (e.g. characteristics relating to structure of training/supervision, delivery). If this information is not explicit in included studies, we will consult protocol/intervention design papers.

We will extract the following components into a separate ‘intervention components’ table:

- Theoretical framework of the intervention (i.e. type of therapy, as categorised above)
- Description of the intervention (including four a priori components of interest: assessment of problems and goals, formulation, homework, and active change strategies [41])
- Intervention setting
- Number of centres providing intervention
- Individual who delivered treatment (e.g. clinician, counsellor)
- Mode of delivery (e.g. telephone, face to face)
- Intervention intensity (duration of treatment, maximum/minimum/median number of sessions, and length/frequency of sessions)
- Group size (if group-based intervention)
- Whether there was signposting to other services
- Manualisation of intervention (yes/no)
- Fidelity to treatment protocol (measured/not measured)

### Critical appraisal of included studies

Risk of bias and study quality will be assessed at study level using two quality appraisal tools. Two reviewers (DR and ES) will independently rate each study in terms of quality and risk of bias. Discrepancies will be discussed, and if not resolved between the two reviewers, JP will help to make a final decision. Two different tools will be used to evaluate quantitative studies and economic evaluation studies.

To assess the risk of bias and study quality in quantitative studies, we will use the Effective Public Health Practice Project’s Quality Assessment Tool for Quantitative Studies [42]. This tool was designed to appraise a number of different study types, including randomised controlled trials (RCTs), controlled clinical trials (CCTs), cohort analytic studies, case-control studies, cohort studies, and interrupted time series studies. It is an accepted tool for the use of systematic reviews of intervention effectiveness [43]. The tool contains eight different sections, each with multiple questions: selection bias, study design, confounders, blinding, data collection methods, withdrawals and drop-outs, intervention integrity, and analyses. Each section receives a score of 1 (strong), 2 (moderate), or 3 (weak), and a final score is determined by the number of 'weak' ratings.

Economic studies will be assessed using the Drummond checklist [44], as recommended by the Cochrane Handbook for Systematic Reviews of Interventions [45]. The Drummond checklist contains 35 questions with four answers each (‘yes,’ ‘no,’ ‘not clear,’ and ‘not appropriate), divided into three categories: study design, data collection, and analysis and interpretation of results. There is no final score assigned to papers, so quality will be reported in narrative style.

### Data synthesis and analysis

#### Narrative synthesis

When presenting results from uncontrolled studies (e.g. pre-post designs, cohort studies), we will use Popay and colleagues’ guidance for narrative synthesis [46]. We will further use this guidance to report the common components of effective interventions. The process may involve the following elements, where feasible and appropriate:

- Develop a synthesis of included studies’ findings (i.e. describe patterns across studies)
- Explore relationships between the findings (i.e. examine factors related to intervention effects)
- Assess the overall robustness of the evidence as a whole (i.e. comment on the generalisability of findings) [46]

Our narrative synthesis will inform Research Question 1 by:

- Synthesising comparative findings from controlled studies not included in meta-analyses (e.g. where there are not enough studies to conduct meta-analysis)
- Examining changes from baseline to follow-up measurements in any of our outcomes of interest for all studies

The synthesis will also address Research Question 2 by:

- Providing a qualitative account of our four a priori components of interest—assessment of problems and goals, formulation, homework, and active change strategies [41]—in relation to intervention effect for studies not included in our meta-analyses
- Providing a qualitative account of any other variables from our components table in relation to the intervention effect for all studies

#### Economic analysis

Economic studies will be presented in tables describing the study characteristics and study results. These will be described in a narrative approach and will include discussion of study quality as well as discussion of study homogeneity. Because cost-effectiveness of one intervention compared with another is heavily dependent on the methods of economic evaluation used in identified studies, decision rules [47] will vary dependent on the

- Method of economic evaluation selected (e.g. cost-effectiveness analysis, cost-utility analysis, cost-benefit analysis, etc.),
- Outcome measure selected (e.g. QALYs, DALYs, clinical measures, etc.), and
- Study setting (different countries have different thresholds an intervention must achieve to be considered cost-effective, so whilst in England, the threshold is £20,000–£30,000 per QALY, the threshold will be different in different countries and for different currencies).

#### Meta-analysis

If two or more RCT/CCT studies report on a given outcome of interest, we will address Research Question 1 by quantitatively synthesising the results via random-effects meta-analysis (we assume a priori that there will be heterogeneity between the included studies; random-effects estimates of effect size are therefore more conservative). Separate meta-analyses will be conducted per outcome. We will compare studies on the basis of the framework of psychological intervention and will group controls into the following categories: psychological therapies/interventions and treatment as usual (we will not include pharmacological or dietary interventions, as these would not align with our aims or inclusion/exclusion criteria). If possible, we will perform subgroup analyses by clinical versus non-clinical study populations, as they may feature differing thresholds in terms of inclusion.

To answer Research Question 2, we will further perform subgroup analyses by our four a priori components of interest, given that there are at least two studies in each comparison group (i.e. two studies *with* the component, and two studies *without* the component).

Our outcomes will be assessed as follows:

- Our primary outcome, i.e. the proportion of participants achieving remission/recovery from psychotic experiences, will be analysed through calculation of an odds ratio (OR) with a 95% confidence interval (CI).
- Our secondary outcomes, i.e. changes in positive/negative psychotic symptoms, distress, functioning, depression, anxiety, and quality of life, will be analysed using standardised mean differences (with 95% CIs).

Where available, we will use intention-to-treat data in our analysis. We will not use meta-analysis to examine outcome data from uncontrolled studies or economic data.

#### Assessment of heterogeneity

We will consider clinical heterogeneity [45] (e.g. diversity of intervention frameworks) before conducting our meta-analysis, to help ensure this method is appropriate for our data. If meta-analysis is appropriate, we will quantitatively assess heterogeneity using a chi-squared test, *Q* statistic, and *I*^2^ statistic. We will use the approximate guidelines suggested by Higgins and colleagues [48], which state that *I*^2^ values of 0%, 25%, 50%, and 75% represent no, low, moderate, and high heterogeneity, respectively.

#### Sensitivity analysis

We will perform sensitivity analyses within the meta-analysed studies to explore the effect of studies with a high risk of bias (e.g. studies that do not use intention-to-treat reporting, studies with high attrition, and studies with missing data). We will do this by removing those studies that received a ‘high’ risk of bias rating in *any* of the bias sections of the Effective Public Health Practice Project Quality Assessment Tool from the meta-analysis and re-computing a pooled estimate of intervention effect. We will further conduct an analysis to assess the impact of removing CCTs on the pooled effect estimate, i.e. using only RCTs to estimate therapeutic effects.

### Assessment of meta-biases

We will assess bias (e.g. publication bias, language bias, citation bias) through an examination of funnel plot symmetry (if ten or more studies are included in the meta-analysis) and Egger’s test of the intercept [49]. Where study protocols are available, we will examine outcomes reporting bias by comparing outcomes listed in the protocol with those presented in the published report. If appropriate, sensitivity analyses may also be conducted to determine the impact of outcomes reporting bias on meta-analytic results.

### Confidence in cumulative evidence

In order to assess the confidence in the cumulative evidence, we will use the GRADE approach, developed by the Grading of Recommendations Assessment, Development and Evaluation working group [50]. The GRADE assessment method covers the following domains: study design, study quality, consistency, and directness [50]. As recommended by the GRADE Working Group, we will begin by ranking according to study design (randomised trials are rated ‘high,’ observational studies ‘low’). We will then downgrade for study quality limitations (measured by our quality rating tools), inconsistency (measured by unexplained inconsistency in results), uncertainty about directness, imprecise or sparse data, or likelihood of reporting bias (if possible to measure). We will upgrade for strong evidence of association or indication of dose-response effects. We will also upgrade if adjustment for all plausible residual confounders would likely have *reduced* the magnitude of observed intervention effect [50]. In making final assessments, we will use the GRADE working group’s recommendations of four levels of evidence quality: high, moderate, low, and very low [51].

## Discussion

Available evidence suggests that psychotic experiences are often present in individuals with common mental disorders such as anxiety and depression, and that rather than solely indicating an imminent transition to a psychotic disorder, they are frequently indicators of severity of common mental distress. Many of these individuals seek help in general care settings, such as IAPT services within primary care, where psychotic experiences are neither measured nor treated. This review will provide an evidence synthesis of the effectiveness and cost-effectiveness of any psychological interventions used for the treatment of psychotic experiences. The identification of specific components associated with intervention effectiveness will augment existing evidence that can inform the development of a new, tailored intervention to improve outcomes related to psychotic symptoms, anxiety and depression, distress, functioning (including social, occupational, and academic), and quality of life. To the authors’ knowledge, this is the first systematic review that explicitly and exclusively intends to inform the development of therapeutic interventions to ameliorate psychotic experiences and their associated distress.

## Appendix

**Table 1:** Search strategy for PsycINFO

S1	sub-clinical or non-psychotic or ((“high risk*” or “clinical risk*” or “at risk”) N5 psychosis) or (prodrom*) or “at risk mental state” or subthreshold or “pre-psychotic” or “psychotic-like”
S2	psychotic or vision* or voice* or hallucinat* or “see* thing*” or paranoi* or psychosis or delusion* or psychopatholog* or percept* or suspicious* or “anomalous experiences” or “unusual experiences” or “atypical experiences” or “abnormal experiences” or “dissociation”
S3	“psychological treatment*” or cbt or “cognitive behav* therap*” or “psychological intervention*” or “cognitive therap*” or emdr or “eye movement desensiti?ation reprocessing” or emdt or “eye movement desensiti?ation therapy” or therap* or psychosocial or psychoeducation* or “family-focused” or “meta-cognitive therapy” or “meta-cognitive treatment” or “trans-diagnostic treatment” or “trans-diagnostic therapy”
S4	((DE “Cognitive Therapy”) OR (DE “Cognitive Behavior Therapy” OR DE “Acceptance and Commitment Therapy”)) OR (DE “Psychotherapy” OR DE “Adlerian Psychotherapy” OR DE “Adolescent Psychotherapy” OR DE “Affirmative Therapy” OR DE “Analytical Psychotherapy” OR DE “Autogenic Training” OR DE “Behavior Therapy” OR DE “Brief Psychotherapy” OR DE “Brief Relational Therapy” OR DE “Child Psychotherapy” OR DE “Client Centered Therapy” OR DE “Cognitive Behavior Therapy” OR DE “Conversion Therapy” OR DE “Eclectic Psychotherapy” OR DE “Emotion Focused Therapy” OR DE “Existential Therapy” OR DE “Experiential Psychotherapy” OR DE “Expressive Psychotherapy” OR DE “Eye Movement Desensitization Therapy” OR DE “Feminist Therapy” OR DE “Geriatric Psychotherapy” OR DE “Gestalt Therapy” OR DE “Group Psychotherapy” OR DE “Guided Imagery” OR DE “Humanistic Psychotherapy” OR DE “Hypnotherapy” OR DE “Individual Psychotherapy” OR DE “Insight Therapy” OR DE “Integrative Psychotherapy” OR DE “Interpersonal Psychotherapy” OR DE “Logotherapy” OR DE “Narrative Therapy” OR DE “Network Therapy” OR DE “Persuasion Therapy” OR DE “Primal Therapy” OR DE “Psychoanalysis” OR DE “Psychodrama” OR DE “Psychodynamic Psychotherapy” OR DE “Psychotherapeutic Counseling” OR DE “Rational Emotive Behavior Therapy” OR DE “Reality Therapy” OR DE “Relationship Therapy” OR DE “Solution Focused Therapy” OR DE “Supportive Psychotherapy” OR DE “Transactional Analysis”)
S5	S3 OR S4
S6	(((DE “Hallucinations” OR DE “Auditory Hallucinations” OR DE “Drug Induced Hallucinations” OR DE “Hypnagogic Hallucinations” OR DE “Visual Hallucinations”) OR (DE “Auditory Hallucinations”)) OR (DE “Paranoia” OR DE “Paranoia (Psychosis)” OR DE “Folie A Deux”)) OR (DE “Psychosis” OR DE “Acute Psychosis” OR DE “Affective Psychosis” OR DE “Alcoholic Psychosis” OR DE “Capgras Syndrome” OR DE “Childhood Psychosis” OR DE “Chronic Psychosis” OR DE “Experimental Psychosis” OR DE “Hallucinosis” OR DE “Paranoia (Psychosis)” OR DE “Postpartum Psychosis” OR DE “Reactive Psychosis” OR DE “Schizophrenia” OR DE “Senile Psychosis” OR DE “Toxic Psychoses”)
S7	S2 OR S6
S8	(DE “Prodrome”) OR (DE “At Risk Populations”)
S9	S1 OR S8
S10	S5 AND S7 AND S9

Filters: 2000–2018

## Abbreviations

CAARMS: Comprehensive Assessment of At-Risk Mental States
CBT: Cognitive behavioural therapy
CCT: Controlled clinical trial
CHR: Clinical high risk
CI: Confidence interval
DALYs: Disability-adjusted life years
FEP: First episode psychosis
IAPT: Improving Access to Psychological Therapies
NICE: National Institute for Health and Care Excellence
NIHR: National Institute for Health Research
QALYs: Quality-adjusted life years
UHR: Ultra-high risk
